# Supplementary material for: Changes in temperature perception in transgender persons undergoing gender-affirming hormone therapy
Source: Commun Med (Lond). 2026 Feb 7;6:146. doi: 10.1038/s43856-026-01420-0 (PMC12993048; doi:10.1038/s43856-026-01420-0)
Supplement: Supplementary file 2 — Supplement [file 43856_2026_1420_MOESM2_ESM.pdf]

Supplemental Material

Supplemental Methods

BEFORE QUANTITATIVE SENSORY TESTING

To be completed by the investigator:

- Room temperature (in °C):
- Skin temp. before warming up the hands (in °C, in the same region of the hand where the probe will be placed; if <28 °C: Hold subject's hands under warm water for 20 seconds):
- Skin temperature after warming up (in °C, question can be skipped for temperatures >28 °C):

Completed by the test person:

How would you rate the CURRENT ROOM TEMPERATURE?

Very cold										Very warm
0	1	2	3	4	5	6	7	8	9	10

Scale for all following questions:

Not at all										often
0	1	2	3	4	5	6	7	8	9	10

I feel WARM in the following situations: When I am sitting quietly (e.g. at the computer, watching TV, reading).

I feel WARM in the following situations: During daily activities.

I feel WARM in the following situations: While I am at home.

I feel WARM in the following situations: While I am in a building or room but not at home.

I feel WARM in the following situations: When I concentrate hard.

I feel WARM in the following situations: In bed.

I feel WARM in the following situations: When I am outside.

I feel COLD in the following situations: When I am sitting quietly (e.g. at the computer, watching TV, reading).

I feel COLD in the following situations: During daily activities.

I feel COLD in the following situations: While I am at home.

I feel COLD in the following situations: While I am in a building or room but not at home.

I feel COLD in the following situations: In bed.

I feel COLD in the following situations: When I'm outside.

I feel COLD in the following situations: When I concentrate hard.

I feel a sudden heat rising inside me.

I feel a sudden cold rising inside me.

- Skin temperature (in °C) at the location on the hand where the probe was placed:

Suppl. Figure S1: Temperature perception questionnaire. Questions of the temperature perception questionnaire (translated from German [original language] to English) based on "The experienced temperature sensitivity and regulation survey" (Van Someren et al.). Summary Score Warm = Sum of scores of items 2+3+4+5+6+7+8. Summary Score Cold = Sum of scores of items 9+10+11+12+13+14+15.

<u>*Supplemental Results*</u>

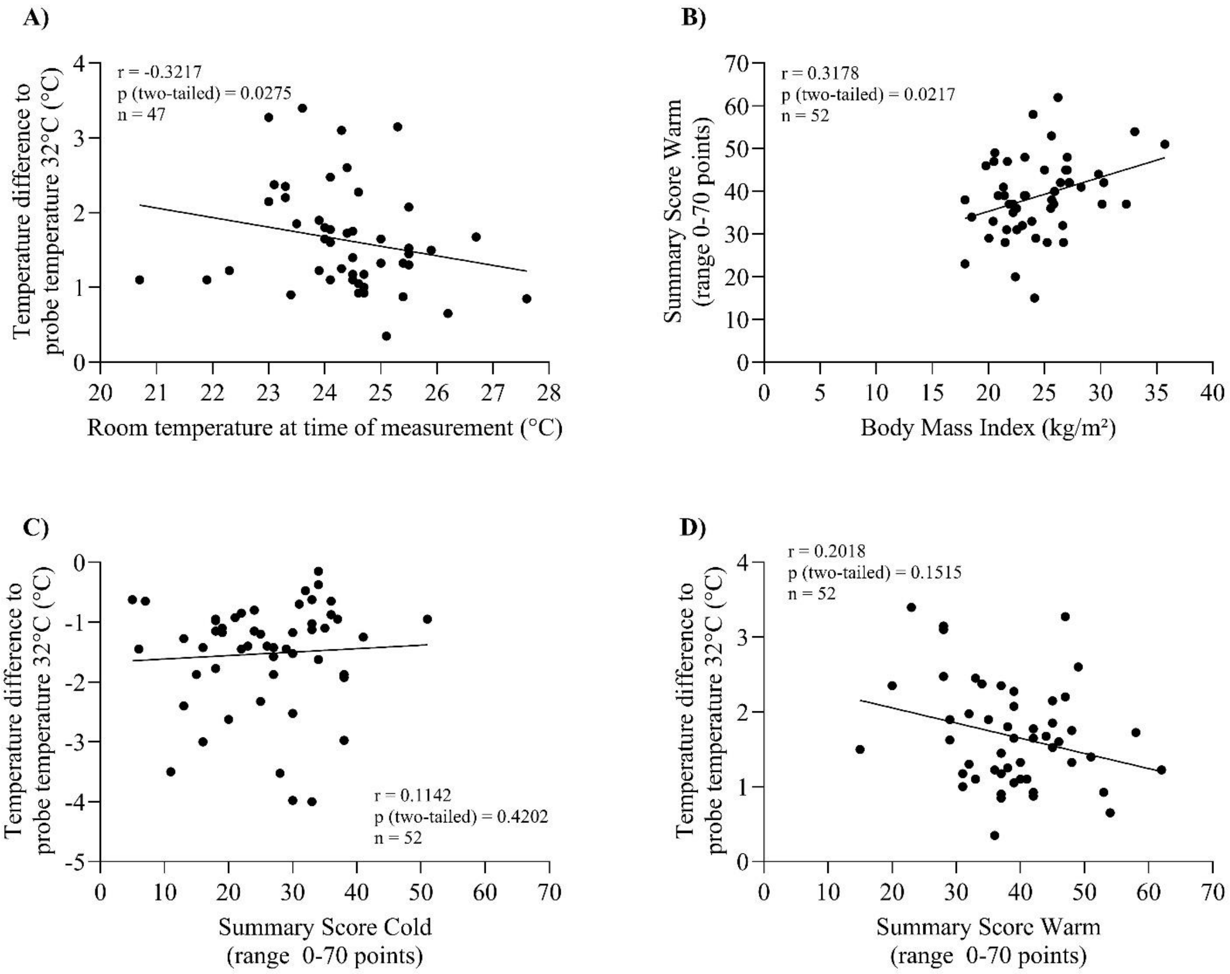

Suppl. Figure S2: Additional Spearman correlations I. Spearman correlations of (**A**) temperature detection threshold "warm" and room temperature, (**B**) summary score warm and body mass index, (**C**) temperature detection threshold "cold" and summary score cold and (**D**) temperature detection threshold "warm" and summary score warm.

Suppl. Table S1: 90% confidence intervals of equivalence paired samples t-tests

	0 vs. 3 months	0 vs. 6 months	3 vs. 6 months
Cold detection threshold			
Trans women	-1.030 to -0.010	-1.124 to -0.021	-0.270 to 0.474
Trans men	-0.241 to 0.188	-0.360 to 0.037	-0.227 to 0.150
Cis women	-0.872 to 0.222	-0.973 to 0.283	0.132 to 0.207
Cis men	-0.985 to -0.071	-0.944 to 0.177	-0.301 to 0.613
Warm detection threshold			
Trans women	-0.048 to 0.649	-0.171 to 0.957	-0.368 to 0.402
Trans men	-0.243 to 0.311	-0.353 to 0.419	-0.210 to 0.210
Cis women	0.050 to 0.454	-0.508 to 0.435	-0.650 to 0.170
Cis men	-0.004 to 0.549	-0.126 to 0.787	-0.357 to 0.476
Cold Pain			
Trans women	-3.941 to 2.141	-7.515 to 0.815	-1.586 to 2.941
Trans men	-6.265 to 0.278	-5.060 to 4.096	-0.744 to 4.907
Cis women	-6.983 to 2.655	-7.226 to 1.359	-5.082 to 0.038
Cis men	-7.890 to 16.490	-9.195 to 5.195	-6.679 to 0.512
Heat Pain			
Trans women	-1.459 to 2.439	-0.642 to 0.882	-2.386 to 0.831
Trans men	-2.477 to 0.824	-1.104 to 2.021	-0.256 to 3.511
Cis women	-2.725 to 1.979	-1.166 to 2.655	-0.379 to 2.424
Cis men	-0.549 to 5.321	0.378 to 2.222	-4.488 to 0.459
Summary Score Cold			
Trans women	-8.987 to 1.654	-12.637 to -1.000	-7.586 to 2.495
Trans men	-5.490 to 4.902	-3.211 to 8.442	-5.867 to 7.251
Cis women	-4.672 to -0.328	-8.009 to 0.191	-5.117 to 4.717
Cis men	-8.189 to 6.633	-4.723 to 6.723	-3.743 to 8.493
Summary Score Warm			
Trans women	-0.466 to 7.299	-4.201 to 4.383	-7.666 to -0.334
Trans men	-5.923 to 2.041	-6.866 to 3.482	-3.652 to 6.729
Cis women	-0.592 to 6.759	-2.891 to 4.163	-8.935 to 1.735
Cis men	-7.217 to 2.328	-3.951 to 5.506	0.264 to 9.736

Equivalence paired t-tests with raw equivalence bounds (−0.05 to 0.05), $\alpha = 0.05$; equivalence is inferred if the 90% confidence interval is fully contained within the bounds.

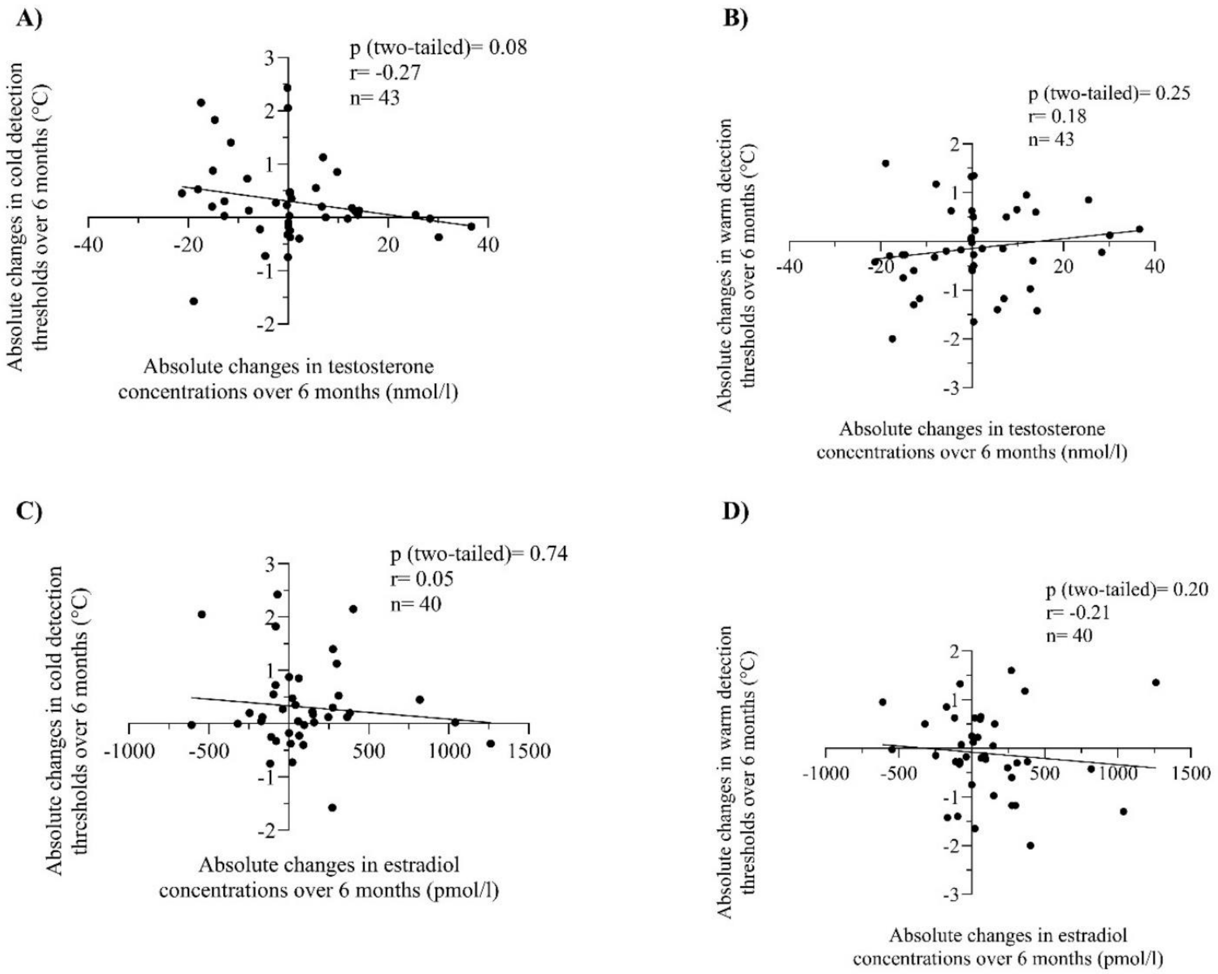

Suppl. Figure S3: Additional Spearman correlations II. Spearman correlations of absolute changes over 6 months in (**A**) temperature detection thresholds cold and serum testosterone concentrations, (**B**) temperature detection thresholds warm and serum testosterone concentrations, (**C**) temperature detection thresholds cold and serum estradiol concentrations, and (**D**) temperature detection thresholds warm and serum estradiol concentrations.

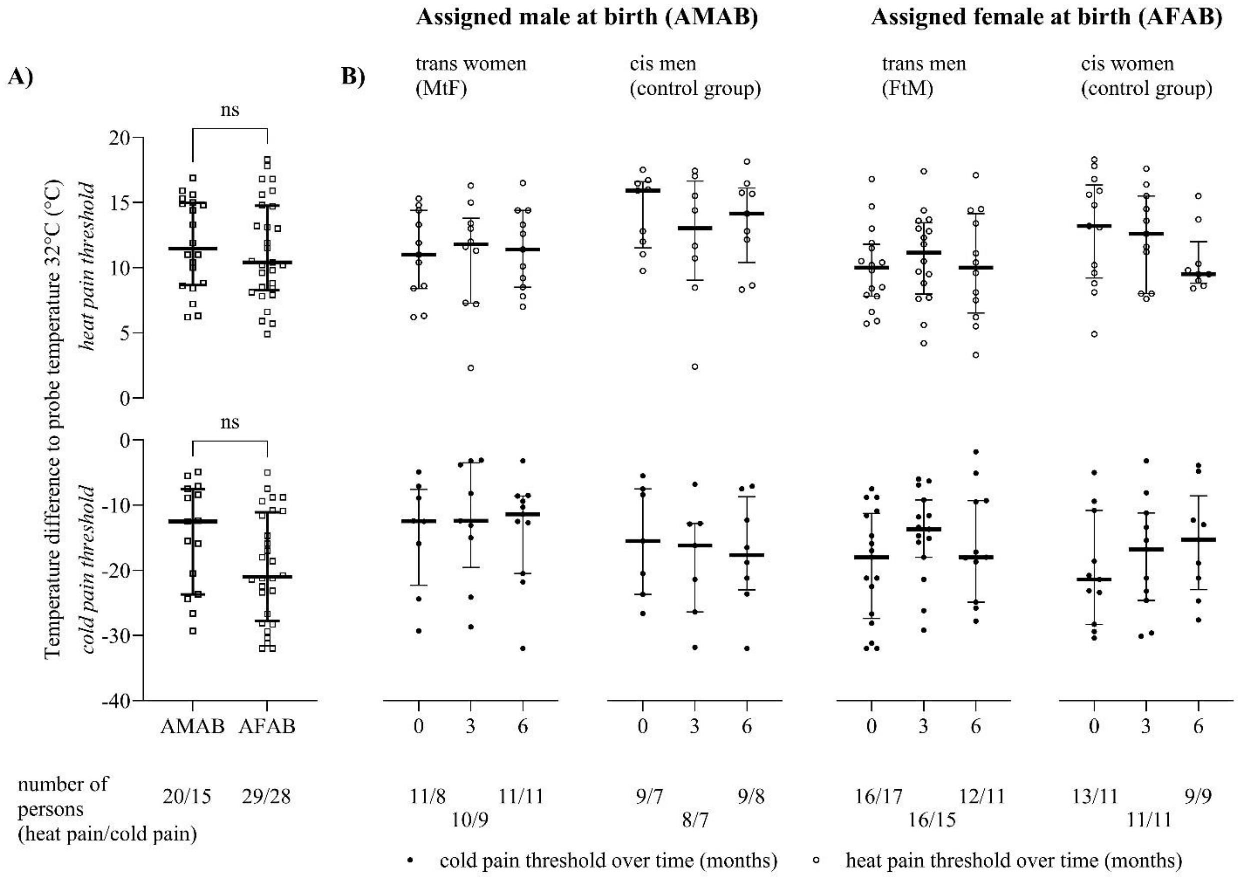

Suppl. Figure S4: Heat and cold pain thresholds measured by quantitative sensory testing. Median + interquartile range of cold and heat pain thresholds measured with quantitative sensory testing (**A**) grouped by assigned sex at birth and (**B**) in trans women, cis men, trans men, and cis women. Statistical testing: (**A**) Mann-Whitney-U test, (**B**) Durbin-Skillings-Mack test. Assigned female at birth: all data from trans men before GAHT and cis women at baseline, assigned male at birth: all data from trans women before GAHT and cis men at baseline, FtM: female-to-male transgender, GAHT: gender-affirming hormone therapy, MtF: male-to-female transgender, ns: $p>0.05$.

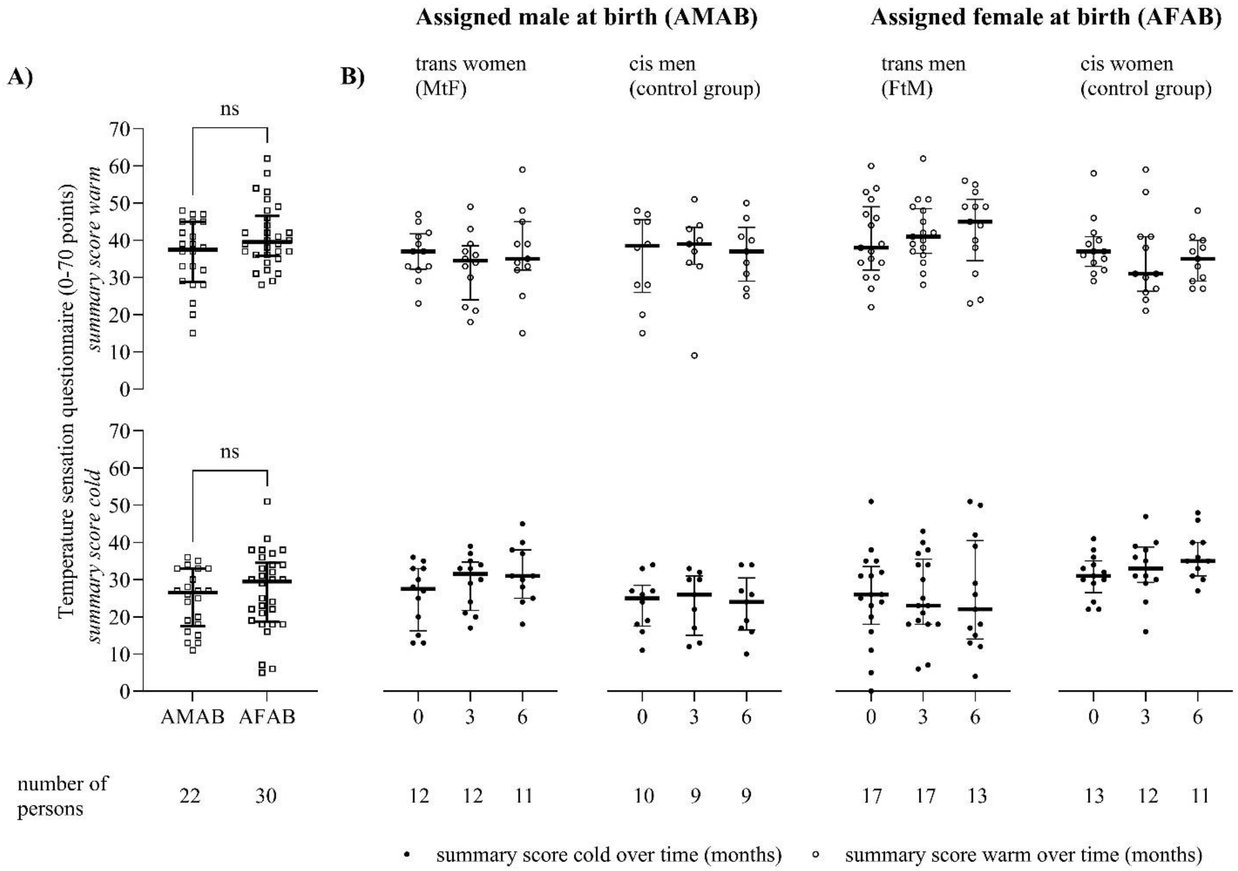

Suppl. Figure S5: Scores of temperature sensation questionnaires. Median + interquartile range of summary scores of temperature sensation questionnaire (**A**) grouped by assigned sex at birth and (**B**) in trans women, cis men, trans men, and cis women. Statistical testing: (**A**) Mann-Whitney-U test, (**B**) Durbin-Skillings-Mack test. Assigned female at birth: all data from trans men before GAHT and cis women at baseline, assigned male at birth: all data from trans women before GAHT and cis men at baseline, FtM: female-to-male transgender, GAHT: gender-affirming hormone therapy, MtF: male-to-female transgender, ns: p>0.05.

Additional analyses of subjective temperature perception and the occurrence of cold and hot flushes with Durbin-Skillings-Mack test

There was no difference in the evaluation of the current room temperature between sexes assigned at birth (p=0.41). Trans women evaluated the current room temperature higher after 6 months of treatment with gender-affirming hormone therapy (GAHT) (p=0.049, *post-hoc*: non-

significant in Conover's procedure). We found no changes in the evaluation of the current room temperature in trans men ($p=0.79$), cis men ($p=0.70$), or cis women ($p=0.72$).

We did not find differences in the occurrence of cold and hot flushes as reported by questionnaires at baseline between persons assigned female and male at birth ($p=0.95$ and $p=0.06$). Trans men reported over the period of 6 months of GAHT an increase in sudden feelings of coldness ($p=0.03$, *post-hoc*: non-significant in Conover's procedure), but no changes in hot flushes ($p=0.56$). No changes in sudden feelings of coldness and heat were observed in trans women ($p=0.77$ and $p=0.56$), cis men ($p=0.24$ and $p=0.32$), and cis women ($p=0.94$ and $p=0.55$).

Results calculated with Friedman test (persons with missing data excluded)

Testosterone and estradiol concentrations

Four cis women who were treated with hormonal contraceptives with ethinylestradiol during the measurements were excluded from the analysis of estradiol concentrations. As expected, testosterone serum concentrations decreased significantly in trans women during 6 months of GAHT ($p\leq0.0001$, Dunn's Test 0 vs. 3 months $p=0.008$, 0 vs. 6 months $p=0.0002$, 3 vs. 6 months $p=0.86$) and estradiol concentrations increased significantly ($p=0.0006$, Dunn's Test 0 vs. 3 months $p=0.003$, 0 vs. 6 months $p=0.02$, 3 vs. 6 months $p>0.99$). In trans men, testosterone concentrations increased significantly ($p\leq0.0001$, Dunn's Test 0 vs. 3 months $p=0.0003$, 0 vs. 6 months $p=0.0006$, 3 vs. 6 months $p>0.99$), while we did not find changes in serum estradiol concentrations ($p=0.79$). We did not find changes in testosterone concentrations in cis men and cis women ($p=0.53$ and $p=0.81$), neither in estradiol concentrations ($p=0.79$ and $p=0.74$) (Figure 1).

Quantitative sensory testing for temperature sensation

In trans women, over the period of six months of GAHT, the thresholds for both cold (p=0.0008, Dunn's Test 0 vs. 3 months p=0.002, 0 vs. 6 months p=0.02, 3 vs. 6 months p>0.99) and warm (p=0.046, Dunn's Test 0 vs. 3 months p=0.35, 0 vs. 6 months p=0.04, 3 vs. 6 months p>0.99) detection decreased significantly. We found no significant changes in temperature detection thresholds in trans men (p=0.47 and p=0.79), cis women (p=0.90 and p=0.32), or cis men (p=0.24 and p=0.12) (Figure 2).

We found no significant changes in the temperature associated with cold pain or heat pain in any of the four study groups over the study period (Suppl. Figure S4).

Temperature perception questionnaire

For the summary scores cold and warm, over the period of six months of GAHT, neither in trans women (p=0.12 and p=0.22), nor trans men (p=0.12 and p=0.74), nor cis men (p=0.71 and p=0.53), nor cis women (p=0.32 and p=0.19), we found statistically significant changes.

Trans men reported over the period of 6 months of GAHT an increase in sudden feelings of coldness (p=0.02, Dunn's Test 0 vs. 3 months p=0.60, 0 vs. 6 months p=0.60, 3 vs. 6 months p=0.03), but no changes in hot flushes (p=0.46). No significant changes in sudden feelings of coldness and heat were detected in trans women (p=0.40 and p=0.58), cis men (p=0.18 and p=0.28), or cis women (p=0.94 and p=0.47).

Trans women evaluated the current room temperature higher after 6 months of treatment with GAHT (p=0.0129, Dunn's Test 0 vs. 3 months p>0.99, 0 vs. 6 months p=0.40, 3 vs. 6 months p=0.057). We found no changes in the evaluation of the current room temperature in trans men (p=0.66), cis men (p=0.74), or cis women (p=0.91).
