# Supplementary material for: Changes in temperature perception in transgender persons undergoing gender-affirming hormone therapy
Source: Commun Med (Lond). 2026 Feb 7;6:146. doi: 10.1038/s43856-026-01420-0 (PMC12993048; doi:10.1038/s43856-026-01420-0)
Supplement: Supplementary file 3 — Description of Additional Supplementary Data [file 43856_2026_1420_MOESM3_ESM.docx]

Description of additional supplementary file

File name: Supplementary Dataset 1

Description: Numerical results underlying Fig. 1 and 2
